# Supplementary material for: Breastfeeding self-efficacy status and associated factors among postpartum mothers at Hadiya Zone public hospitals, Southern Ethiopia
Source: PLoS One. 2025 Feb 10;20(2):e0317763. doi: 10.1371/journal.pone.0317763 (PMC11809790; doi:10.1371/journal.pone.0317763)
Supplement: S1 Questionnaire — (ZIP) [file pone.0317763.s001.zip › S1 Questionnaire(Socio demographic).docx]

S1 Table: socio-demographic characteristics and obstetrics related variables

| S.N | QUESTION | RESPONSE | Remark |
| --- | --- | --- | --- |
| 101 | How old are you | 1. <25 2. 25-35 3. >35 |  |
| 102 | What is your educational level | 1. No formal education 2. Primary school(1-8) 3. Secondary school(9-12) 4. Tertiary |  |
| 103 | What is your marital status | 1. Unmarried 2. Married |  |
| 104 | What is your occupation | 1. Government employer 2. Self employed 3. Housewife |  |
| 105 | What is your husband educational level | 1. No formal education 2. Primary(1-8) 3. Secondary(9-12)and above |  |
| 106 | What is your husband occupation | 1. Government employer 2. Merchant 3. Self-employed 4. Unemployed 5. Harmer 6. Other specify |  |
| 107 | What is your residence | 1. Urban 2. Rural |  |
| 108 | What is your religion | 1. Orthodox 2. Protestant 3. Muslim 4. Catholic 5. Other/specify |  |
| Obstetrics related questions | | | |
| 301 | Number of parity | 1. Primi Para 2. Multi para |  |
| 302 | Have you attended Antenatal clinic in any health facility while you were pregnant in the current pregnancy? | 1. Yes 2. No | If No203,204 |
| 303 | How many times you attend(visit) antenatal care in that clinic | 1. 1-3 2. 4+ |  |
| 304 | Have you ever been informed/learned about breastfeeding while you were pregnant in ANC follow up in current pregnancy | 1. Yes 2. No |  |
| 305 | What was the current pregnancy intention with the last child? | 1. intended 2. Unintended |  |
| 306 | What is the gestational age of the new born in current delivery | 1. Pri Term 2. Post term 3. Term |  |
| 307 | How many children do you have now? | _______ |  |
| 308 | What was mode of delivery | 1. Normal spontaneous vaginal delivery(N.S.V.D) 2. Caesarean section(C/S) |  |
| 309 | What is the sex of your current baby | 1. Male 2. Female |  |
| 310 | Do you have breast feeding experience | 1. Yes 2. No |  |
