# Supplementary material for: Breastfeeding self-efficacy status and associated factors among postpartum mothers at Hadiya Zone public hospitals, Southern Ethiopia
Source: PLoS One. 2025 Feb 10;20(2):e0317763. doi: 10.1371/journal.pone.0317763 (PMC11809790; doi:10.1371/journal.pone.0317763)
Supplement: S1 Questionnaire — (ZIP) [file pone.0317763.s001.zip › S1 BFSE-SF(English version).docx]

S1 Table: - mother’s postnatal breast feeding self-efficacy scale.

For each of the following statements, please choose the answer that best describes how confident you are with breastfeeding your new baby. Please mark your answer by circling the number that is closest to how you feel. There is no right or wrong answer.

1. Not at all confident
2. Sometimes confident
3. neutral
4. Confident
5. Very confident

| No | Question | Response | | | | |
| --- | --- | --- | --- | --- | --- | --- |
| 201 | I Determine that my baby is getting enough milk | 1 | 2 | 3 | 4 | 5 |
| 202 | I Successfully cope with breastfeeding like I have with other challenging tasks | 1 | 2 | 3 | 4 | 5 |
| 203 | I Breastfeed my baby without using formula as a supplement | 1 | 2 | 3 | 4 | 5 |
| 204 | I Ensure that my baby is properly latched on for the whole feeding | 1 | 2 | 3 | 4 | 5 |
| 205 | I Manage the breastfeeding situation to my satisfaction | 1 | 2 | 3 | 4 | 5 |
| 206 | I Manage to breastfeed even if my baby is crying | 1 | 2 | 3 | 4 | 5 |
| 207 | I Keep wanting to breastfeed | 1 | 2 | 3 | 4 | 5 |
| 208 | I Comfortably breastfeed with my family members present | 1 | 2 | 3 | 4 | 5 |
| 209 | I Be satisfied with my breastfeeding experience | 1 | 2 | 3 | 4 | 5 |
| 210 | I Deal with the fact that breastfeeding can be time-consuming | 1 | 2 | 3 | 4 | 5 |
| 211 | I Finish feeding my baby on one breast before switching to the other breast | 1 | 2 | 3 | 4 | 5 |
| 212 | I Continue to breastfeed my baby for every feeding | 1 | 2 | 3 | 4 | 5 |
| 213 | I Manage to keep up with my baby’s breastfeeding demands | 1 | 2 | 3 | 4 | 5 |
| 214 | I Tell when my baby is finished breastfeeding | 1 | 2 | 3 | 4 | 5 |
