# Supplementary material for: Breastfeeding self-efficacy status and associated factors among postpartum mothers at Hadiya Zone public hospitals, Southern Ethiopia
Source: PLoS One. 2025 Feb 10;20(2):e0317763. doi: 10.1371/journal.pone.0317763 (PMC11809790; doi:10.1371/journal.pone.0317763)
Supplement: S1 Questionnaire — (ZIP) [file pone.0317763.s001.zip › S2 BFSE-SF(Amharic version).docx]

**ክፊል2፡ጡት የማጥባትን ራስን የመቻል የሀሳብን መጠን መለኪያ - አጭር ቅጽ**

ለእያንዳንዱ የሚከተሉት መግለጫዎች፣ እባክዎን አዲሱን ልጅዎን ጡት በማጥባት ምን ያህል በራስ መተማመን እንዳለዎት በተሻለ የሚገልፀውን መልስ ይምረጡ። እባክዎን ለሚሰማዎት ስሜት ቅርብ የሆነውን ቁጥር በመዞር መልስዎን ምልክት ያድርጉበት። ትክክለኛ ወይም የተሳሳተ መልስ የለም.

1 = በፍፁም አለመተማመን 2 = በጣም በራስ አለመተማመን 3 = ክየቱምአይደለሁም 4 = በራስ መተማመን 5 = በጣም በራስ መተማመን

| ተቁ | ጥያቄ | ምላሽ | | | | |
| --- | --- | --- | --- | --- | --- | --- |
| 1 | ልጄ በቂ ወተት እያገኘ መሆኑን ሁልጊዜ ማወቅ እችላለሁ | 1 | 2 | 3 | 4 | 5 |
| 2 | እንደ ሌሎች ፈታኝ ስራዎች ጡት በማጥባት ሁሌም በተሳካ ሁኔታ መቋቋም እችላለሁ | 1 | 2 | 3 | 4 | 5 |
| 3 | ፎርሙላ እንደ ማሟያ ሳልጠቀም ሁል ጊዜ ልጄን ማጥባት እችላለሁ | 1 | 2 | 3 | 4 | 5 |
| 4 | ሁል ጊዜ ልጄ ለሙሉ መመገብ በትክክል መያዙን ማረጋገጥ እችላለሁ | 1 | 2 | 3 | 4 | 5 |
| 5 | ሁል ጊዜ የጡት ማጥባት ሁኔታን እስከ እርካታ ማስተዳደር እችላለሁ | 1 | 2 | 3 | 4 | 5 |
| 6 | ልጄ እያለቀሰ ቢሆንም ሁልጊዜ ጡት ማጥባት እችላለሁ | 1 | 2 | 3 | 4 | 5 |
| 7 | ሁልጊዜም ጡት ማጥባት መሻቴን መቀጠል እችላለሁ | 1 | 2 | 3 | 4 | 5 |
| 8 | የቤተሰቤ አባላት በሚገኙበት ጊዜ ሁል ጊዜ በምቾት ጡት ማጥባት እችላለሁ | 1 | 2 | 3 | 4 | 5 |
| 9 | በጡት ማጥባት ልምዴ ሁል ጊዜ ረካለሁ | 1 | 2 | 3 | 4 | 5 |
| 10 | ጡት ማጥባት ብዙ ጊዜ የሚወስድ የመሆኑን እውነታ ሁልጊዜ መቋቋም እችላለሁ | 1 | 2 | 3 | 4 | 5 |
| 11 | ወደ ሌላኛው ጡት ከመቀየሬ በፊት ሁል ጊዜ ልጄን በአንድ ጡት ማጥባትን መጨረስ እችላለሁ | 1 | 2 | 3 | 4 | 5 |
| 12 | ሁልጊዜም ልጄን ለእያንዳንዱ አመጋገብ ጡት ማጥባቱን መቀጠል እችላለሁ | 1 | 2 | 3 | 4 | 5 |
| 13 | የልጄን የጡት መጥባት ፍላጎቶች ሁልጊዜ ማሟላት እችላለሁ | 1 | 2 | 3 | 4 | 5 |
| 14 | ልጄ ጡት መጥባት መቼ እንደጨረሰ ሁልጊዜ ማወቅ እችላለሁ | 1 | 2 | 3 | 4 | 5 |
