# Supplementary material for: Breastfeeding self-efficacy status and associated factors among postpartum mothers at Hadiya Zone public hospitals, Southern Ethiopia
Source: PLoS One. 2025 Feb 10;20(2):e0317763. doi: 10.1371/journal.pone.0317763 (PMC11809790; doi:10.1371/journal.pone.0317763)
Supplement: Attachment — Submitted filename: Review comments.docx [file pone.0317763.s003.docx]

Review comments

Thank you dear editors for you’re considering me to review this paper!

I have completed my evaluation of the manuscript with the Manuscript Number: PONE-D-23-34434 entitled as “Breastfeeding self-efficacy status and associated factors among postpartum mothers at Hadiya Zone public hospitals, southern Ethiopia”

It is an interesting topic and well written paper. However, I have the following comments to the authors for improvement:

**Abstract:**

1) In the result section you wrote “secondary and above educational status [AOR = 6.856; 95% CI (2.670-17.603)]” but in your model table it said “More than secondary” consistency problem
2) It is not clear “statistical package for social science on Windows version 24.”

**Background:**

3. Put a reference to this sentence ““If self-efficacy is lacking, people tend to behave ineffectually, even though they know what to do’’ says Albert Bandura.”"

4. put references to this sentence “Finally, breastfeeding self-efficacy status is influenced by sociodemographic, obstetric, and health service-related variables and has not been researched before in postpartum mothers in Ethiopia.”

**Methodology:**

5. Why use p=50% and single population proportion formula, why not double population proportion?

6. How the samples were allocated to four study hospitals?

7. Did you show us Multicolinarity results?

**Result:**

**Discussion:**

8. It needs more revision especially “48% of mothers had a high BFSE status; this result is higher than that of a study carried out in Brazil, where 35% of mothers had high BFSE (24), 43.9% of respondents in China had high BFSE (25), and 43.2% of respondents in Indonesia had high BFSE [18]. The reason might be because of sociocultural and economic variation among the countries. This might be the possible reason for the discrepancy in mothers’ BFSE status.” How we can identify your result is higher than the listed references? Put the 95%CI first?

9. *“Nepal, which was 94% [26]. This variation may be due to the smaller sample size, and the sample was collected after the mothers had been breastfeeding for at least two weeks. Involvement in BF or practice increases the possibility of having a higher status in BFSE [27]. Moreover, in Brazil, 95.3 percent of postpartum mothers have a high BFSE status (28). This variation might be secondary to the purposive sampling technique used and the way of classifying the status of BFSE being different, which was categorized into three.”* It is unclear which study has small sample size, use purposive sampling technioque; the current or previous one and the like…..?

10. *Put a reference to this paragraph “The possible justification might be that elderly women are more likely than younger women to have several children. As a result, this presents a chance to breastfeed repeatedly. This shows that having prior BF experience results in having a high status of BFSE.”*

11. “This outcome is supported by research conducted in western Indonesia [18, 32, 33]” are all reference from Indonenia?

Generally the discussion part needs more clarity and rewriting.

**Conclusion:**

It lacks recommendation.

Your model table is 1/5?

I hope the authors will be properly address the questions.

Finally thank you!!
