# Supplementary material for: Breastfeeding self-efficacy status and associated factors among postpartum mothers at Hadiya Zone public hospitals, Southern Ethiopia
Source: PLoS One. 2025 Feb 10;20(2):e0317763. doi: 10.1371/journal.pone.0317763 (PMC11809790; doi:10.1371/journal.pone.0317763)
Supplement: Attachment — Submitted filename: Comment.docx [file pone.0317763.s004.docx]

**General**

- It would be better for the authors to use line number.
- The study appears to be good, but in order for the paper to be readable, the authors should check the language /grammar throughout the document. And also revise for redundancy.

**Background**

- On the first statement, it is not the correct way of writing term definition including the punctuation
- On the 3^rd^ paragraph, the Authors should rewrite the following statement; *“This shows that a high breastfeeding self-efficacy status has power in breastfeeding, whereas it is a reason for failure to BF if it goes lower [3–5].”*
- The authors’ use of abbreviation and a full term should be consistent throughout the document i.e. the word breastfeeding and BF
- On the 4^th^ paragraph, 2^nd^ line, change the words “*children under five”* to under five children.
- On the 4^th^ paragraph, 2^nd^ statement, what does the statement indicate? It is stating breast feeding causes illnesses. “*However, based on statistics from 130 nations' health surveillance, an additional 166 million episodes of diarrheal illnesses and 9 million instances of pneumonia in children under the age of two were caused by BF”.*

The authors should revise and rewrite

- Better to rewrite the fourth paragraph totally.

**Method**

- The authors should rewrite the statement under the population subtitle and make it clear. They have stated that the source population for the study was postpartum mothers at selected hospitals in the zone, but it also states that all hospitals with in the zone are included in the study.
- On the sampling method, there is unnecessary detail about the non-response rate. “*To compensate the participants who refused or stopped the interview before completion of the questionnaire after they were selected to participate in the study*”
- The authors need to clarify how systematic random sampling technique was implemented for postpartum mothers with in 24hours? It is also known that mothers who gave birth spontaneous vaginal delivery stays mostly for 6 hours at hospitals.
- On the data analysis, line 5, it is better to use the term binary logistic regression rather than bivariate since there is a difference between the two. Including on the table caption
- On the data analysis, line 7, the authors should not use the word predictors since the study was cross sectional. Including on the table caption

**Result**

- While the result was well written, one of the descriptive results under the subtitle obstetric and health service related factors is not in line with result with in the table. Specifically, line 1, which states 72.6% and the table states 75 %
- Also the table number in descriptive result and the table at the end are not in line with each other.

**Discussion**

- On the first and second paragraph a study from Brazil is mentioned with different percentage. The authors should specify the study settings.
- In the second paragraph, second statement, it is better to find another reason for variation among studies rather than small sample size.
- On the second paragraph, line 6, the authors should clarify how a purposive sampling technique can cause a variation among results.
- Rewrite the comparison with the study done in Iran. And also the study participants are not similar (pregnant vs. postpartum)

**Conclusion**

- It is better to rewrite the conclusion
- It is better to include clinical implication and recommendation in line with the result.

**Tables**

- The author should check the table numbering and order
- The table on page 19 should also include the variables that were significant in the bivariable regression but not in the multivariable one. i.e. ANC care visit, parity, etc.
