# Supplementary material for: Breastfeeding self-efficacy status and associated factors among postpartum mothers at Hadiya Zone public hospitals, Southern Ethiopia
Source: PLoS One. 2025 Feb 10;20(2):e0317763. doi: 10.1371/journal.pone.0317763 (PMC11809790; doi:10.1371/journal.pone.0317763)
Supplement: Attachment — Submitted filename: Reviewer response to PLOS ONE (BFE).docx [file pone.0317763.s005.docx]

**Reviewers’ response to editor and author’s comments**

**Title:** ***Breastfeeding self-efficacy status and associated factors among postpartum mothers at Hadiya Zone public hospitals, southern Ethiopia***

Manuscript ID: PONE-D-23-34434

From: Reviewer

To: The editor in chief, PLOS ONE

My sincere gratitude will go out to you for sharing this type of research with the scientific community. It has been a pleasure learning about it.

Background: In general, it should be revised because of poor construction of statements, flow and it can’t convince readers so it needs a thorough revision)

References should not be put in square brackets.

What is the difference between a facility and a health institution?

Why you want to conduct in the selected health facilities

The educational status confidence interval is a bit wider, why?

#### Method section: sampling method, an incomplete kind of phrase. So, you need to fully write it as sample size determination and sampling Procedure. Again, you ought to write as a data collection tool and procedure.

To obtain insights, this research must be conducted on a community level, as we must take into account cultural and contextual factors.

#### Please write formally or follow the journal guideline (data collection) like……Data collection tool and procedure.

#### Who collected your data?

#### Who were your supervisors?

#### Did you conduct a pretest? Nothing is mentioned

#### Again, you fail to narrate the internal consistency of the data collection tool (i.e. reliability).

#### I didn’t get, how you control /assure the quality of the data.

#### As you described in the data processing and analysis section, multicollinearity was checked, how did you check it, and what was the result and interpretation, is any variable that scored above acceptable? if any, please …..

#### Why did you prefer to use p<0.25? why not p<0.20?

Where is the operational definition? We don’t know how you measure it.

Since you have an ordinal dependent variable i.e. the level of breastfeeding efficacy, ordinal logistic regression????? Why dichotomy it?

What is the relationship between breastfeeding self-efficacy and breastfeeding technique?

Why do you want to conduct only public hospitals?

why do you say low the proportion of BFE in your conclusion? What are your comparators? You mention the standard.

Why did you consider P is 50%?

Where is the sampling procedure? I didn’t find even a reference for it.

Result

Please write a full statement of the result section

Avoid using the word “study subject” better to use study participants

The standard Deviation is 5.32 which means there is a data set variability that could be outliers /extreme values/ or other factors therefore I suggest you first check the age distribution whether skewed or not or you might consider transformation or you might conduct a sub-group analysis.

Please summarize it.” Mothers were asked about the educational status of their husbands”

Please merge (put in one table together) Table 1 and Table 2

I am not clear about the result of breastfeeding efficacy because you mentioned in your document to measure it you considered the median but here you mentioned taking a single question from the table so there is incompatibility in answering the dependent variable.

What do you mean by “Bivariable analysis in the binary logistic regression model”?

What is the valid reason for the association between BFE with spontaneous delivery?

What are the strengths and limitations of your study?

**Discussion**

There is a resulting mismatch of 35.6% vs 48% you have to correct it. (there is inconsistency /result incompatibility so it is a major comment).

Did you check Brazil, Indonesia, and China match their inclusion and exclusion criteria?

Frequently you mention “Respondents” Please use other terms or minimize them.

Despite this research is indispensable and contributes to the body of knowledge, your way of writing needs more work. The paper will benefit from professional proofreading and editing to

improve the clarity of the written language
