# Supplementary material for: Breastfeeding self-efficacy status and associated factors among postpartum mothers at Hadiya Zone public hospitals, Southern Ethiopia
Source: PLoS One. 2025 Feb 10;20(2):e0317763. doi: 10.1371/journal.pone.0317763 (PMC11809790; doi:10.1371/journal.pone.0317763)
Supplement: Attachment — Submitted filename: Responses for reviewers .docx [file pone.0317763.s006.docx]

Responses for reviewers

| S.no | Reviewers comment |  |
| --- | --- | --- |
|  | We have removed any funding-related text from the manuscript. Please change the online submission form on our behalf. |  |
|  | **Reviewer** | **Response** |
|  | **Academic Editor** |  |
| 1 | What factors are considered when selecting candidate variables for multivariable logistic regression analysis, and why is a p-value cutoff of 0.25 used? are there any other criteria that should be considered when selecting candidate variables? | Thank you very much for all of your insightful and helpful feedback, as well as the questions you asked. We cannot consider any other criterion to pick candidate variables other than this one. The cutoff point of 0.25 was chosen in order to allow for the computation of more variables in multivariable logistic regression. |
| 2 | I'm curious to learn how you selected the four hospitals for this study from among those available in the zone. | Thank you. We take 100% or all of the hospitals that are available in the zone. The hospitals are all that are available in the zone. |
| 3 | Which specific measure of association was employed to quantify the association between the variables of interest? | Thanks, most of crossectional study use OR. We measure the association between variables with OR |
| 4 | It would be beneficial to ensure consistency in the use of decimal points throughout the text. For instance, the first AOR value is presented with three decimal places, the second with four, and the last with only one, which is inconsistent. To maintain consistency, the AOR values and their corresponding confidence intervals should be formatted with the same number of decimal places. | We have included your helpful comments into the manuscript. Thank you. |
| 5 | The conclusion fails to provide a comprehensive interpretation of the study's main result and does not align with the existing body of knowledge. It also neglects to discuss the factors associated with BFSE and the implications of the research findings. | Thank you for your supportive comment. We rewrite the conclusion part of the manuscript. |
| 6 | Needs a complete revision. The work of discussion must reveal the relative importance of the current findings. Implications of the findings, discussion of the limitations of the study, and guidance on future directions for further research. | Thank you for your constructive comment. The discussion part is revised. |
| **Reviewer #1 Response** | | |
| 1 | Why use p=50% and single population proportion formula, why not double population proportion? | Thank you for this question. To the extent of our searching ability, we did not find a similar study that was conducted in our country, particularly in the study area. |
| 2 | How the samples were allocated to four study hospitals? | Thank you for the question. By considering the average number of postpartum clients in each hospital, |
| 3 | Did you show us Multicoliniarity results? | **Thank you for the question. There was an editorial problem, and the way of writing is not correct on the manuscript, there is no multicollinearity among the independent variables in the coefficient table on VIF.** |
| 4 | This outcome is supported by research conducted in western Indonesia [18, 32, 33]” are all reference from Indonesia? | Thank you for your valuable comment. Reference number 18 is only from Indonesia, and the remaining two are from Iran and Turkey. |
|  | **Reviewer #2** |  |
| 1 | Exclusion criteria is not clear.  what if women gave birth home and referred due to complications. | Thank for the question. Critically ill mothers were excluded. We also corrected the way of writing really thanks. |
| 2 | There are many studies conducted in the same topics but you take 59 % to calculate, could you please justify? | Thanks. Yes, there are studies conducted on the same topic. But to the best of our knowledge, we have not found such a topic in Ethiopia. Due to fear of differences in sociocultural and economic differences among the countries, we use 50%, not 59%. |
| 3 | Again, some hospital is comprehensive other are general and primary. How you easily compare those hospital with different services. | Thanks. We also face difficulty in this regard because the zone has only 4 hospitals, which have different levels; however, the data was collected from similar clients in their postpartum period. In all hospitals, there is no different standard of care among hospitals that is given to postpartum mothers, which can cause differences in the BFSE of mothers in the hospitals. |
| 4 | Who were your data collectors? | Thank you for your constructive guidance. The data was collected by four experienced midwives. It was not mentioned in the manuscript, but we added it. |
| 5 | why media was used? Why not mean? Please justify? | Thanks, Because the population was not distributed normally. |
| 6 | Don't you think that parity and BF experience has Multicoliniarity? | Thanks, Multicollinearity was not detected in our study. In fact, yes, parity increased, and BF experience also increased; however, in our final model, parity was not included. |
|  | **Reviwer#3** |  |
| 1 | On the first statement, it is not the correct way of writing term definition including the punctuation | Thank you for your constructive comment. Ccorrected on the manuscript |
| 2 | On the 3^rd^ paragraph, the Authors should rewrite the following statement; *“This shows that a high breastfeeding self-efficacy status has power in breastfeeding, whereas it is a reason for failure to BF if it goes lower [3–5].”* | We grateful for your insightful feedback. We updated. |
| 3 | The authors need to clarify how systematic random sampling technique was implemented for postpartum mothers with in 24hours? It is also known that mothers who gave birth spontaneous vaginal delivery stays mostly for 6 hours at hospitals? | Thanks for the question. Mothers who gave birth spontaneous vaginal delivery stay for 6 hours at the hospital .The data collected from them within this time interval; however, our study includes mothers who give birth in C/S. From these mothers, the data was collected after 8 hours and before they were discharged. That is why 24 hours was used; however, we corrected our ways of writing thanks. |
| 4 | On the first and second paragraph a study from Brazil is mentioned with different percentage. The authors should specify the study settings. | Thank you for your valuable comment. The difference in percentage is because of two different studies conducted in Brazil however, we specifically mention the study settings thanks. |
| 5 | Rewrite the comparison with the study done in Iran. And also, the study participants are not similar (pregnant vs. postpartum) | Thanks, for your insightful observation. Because of participant disparity, we left it out. |
|  | **Reviewer #4:** |  |
| 1 | What is the difference between a facility and a health institution? | Thank you for all of constructive comments and for the questions too, Facility-a particular area or place including buildings, used for a particular purpose or activity.  Health institution-places that provide health care. They include hospitals, clinics, outpatient care centers, and specialized care centers, such as birthing centers and psychiatric care centers.  However, we used this both terms to talk about health institution inconsistently recently replaced by health institution. |
| 2 | Why you want to conduct in the selected health facilities | Thanks for this question. The hospitals are all of the total hospitals that are readily available within the zone serves the community. |
| 3 | The educational status confidence interval is a bit wider, why? | **Thanks, for rising this question, when we see the educational status, most of the mothers are in the primary and secondary categories; however, among mothers with a more than secondary educational status, the number of mothers is smaller than the others. This may make the CI a bit wider.** |
| 4 | To obtain insights, this research must be conducted on a community level, as we must consider cultural and contextual factors. | Thank you for your valuable suggestion. However, the data has already been collected and the results have been calculated. However, we recommend it for the next researchers. |
| 5 | Who collected your data? Who were your supervisors? | Thank you for this question. The data was collected by four experienced midwives with BSc degree under the supervision of one clinical midwife with MSc degree. |
| 6 | Did you conduct a pretest? Nothing is mentioned | Thanks, Yes, pretesting of the instrument was done before the actual data collection. For this: investigator, supervisors and data collectors were taken a part in a pretest of the survey questionnaire among 5% of the sample size. The Pre-test was conducted in Halaba special zone General Hospital naw mentioned on manuscript |
| 7 | I didn’t get, how you control /assure the quality of the data. | Thank you for asking this question. To assure the quality of data supervisor and principal investigator checked the data collectors on daily basis for completeness and consistence of the filled questionnaires. |
| 8 | As you described in the data processing and analysis section, multicollinearity was checked, how did you check it, and what was the result and interpretation, is any variable that scored above acceptable? if any, please. | Thank you for asking this question. Sorry for the way of writing in the paper and there is no Multicoliniarity among the independent variable. |
| 9 | Why did you prefer to use p<0.25? why not p<0.20? | Thank you for this question. To give more chance for variables to compute each other or increase number of variables candidates for the next step. |
| 10 | Where is the operational definition? We don’t know how you measure it. | Thank you for posing this inquiry. In order to evaluate the dependent variable, we utilized a 14-item Likert scale questionnaire called the BFSE-SF. To categorize participants as having a high BFSE, we established the median as the threshold. Individuals scoring below the median were classified as having low BFSE, while those scoring above the median were deemed to have high BFSE status. It is mentioned on instruments part of the manuscript |
| 11 | Since you have an ordinal dependent variable i.e. the level of breastfeeding efficacy, ordinal logistic regression????? Why dichotomy it? | Thank you for your question. Because the population is not normally distributed. |
| 12 | What is the relationship between breastfeeding self-efficacy and breastfeeding technique? | Thank you for the question. Before performing a certain activity, there should be a self-efficacy towards that activity. Like that, having breastfeeding self-efficacy can aid the mother in being good at breastfeeding techniques, if poor BFSE fails technically because not confident (have self-efficacy). Generally, the status of self-efficacy determines the competency of a certain technique such that BF technique |
| 13 | Why do you want to conduct only public hospitals? | Thanks, Hadiya Zone does not have a private hospital that offers obstetrics services to the general public. |
| 15 | why do you say low the proportion of BFE in your conclusion? What are your comparators? You mention the standard. | Thank you for the question. It is based on the cut of point which is operationalize during the categorization. Not to say based on standards which used as a comparator. |
| 16 | Why did you consider P is 50%? | Thanks for the question. To the extent of our searching skill there is no similar research conducted in Ethiopia. |
| 17 | Please write a full statement of the result section | Thank you for your constructive comment. and result section is revised again |
| 18 | The standard Deviation is 5.32 which means there is a data set variability that could be outliers /extreme values/ or other factors therefore I suggest you first check the age distribution whether skewed or not or you might consider transformation or you might conduct a sub-group analysis. | Thank you. There was no outlier in our data on age; the minimum age was 18 and the maximum was 41. When we run SPSS, the histogram shows no skewness, and age is normally distributed. |
| 19 | What do you mean by “Bivariable analysis in the binary logistic regression model”? | Thank you for your constructive comment. Sorry it is editorial default and it mean to say binary logistic regration. Corrected on document. |
| 20 | What is the valid reason for the association between BFSE with spontaneous delivery? | Thanks a lot for the question. Those who gave birth in spontaneous vaginal delivery were confident because they had no C/S wounds, complications of anesthesia, or breast engorgement problems compared to C/S. Being free from all of these complications increased the mother’s confidence, or BFSE. |
| 21 | What are the strengths and limitations of your study? | Thank you for raising this question. the high response rate to the interviews (98.8%), the data were primary, and multiple variables were used to identify factors associated with BFSE. It has also limitation, since the study is a cross-sectional approach, it has a limitation to formulate a casual effect association. |
| 22 | There is a resulting mismatch of 35.6% vs 48% you have to correct it. (there is inconsistency /result incompatibility so it is a major comment). | Thank you for your inquiry. The percentage, 35.6, indicates that out of the 14 questions, only one item or question received a higher score among participants; the lowest-scoring item among the 14 is 26.4. Table 3 provides the outcomes of the remaining items, which can be found in the descriptive section. However, after evaluating the breastfeeding self-efficacy level of postpartum mothers using a 14-item Likert scale questionnaire, the prevalence of breastfeeding self-efficacy is determined to be 48%. |
| 23 | Did you check Brazil, Indonesia, and China match their inclusion and exclusion criteria? | Thank you for your valuable guidance. We have reviewed and revised the manuscript accordingly. |
