# Supplementary material for: Breastfeeding self-efficacy status and associated factors among postpartum mothers at Hadiya Zone public hospitals, Southern Ethiopia
Source: PLoS One. 2025 Feb 10;20(2):e0317763. doi: 10.1371/journal.pone.0317763 (PMC11809790; doi:10.1371/journal.pone.0317763)
Supplement: Attachment — Submitted filename: Response to Reviewers LN.docx [file pone.0317763.s007.docx]

**Response to Reviewers**

**To:** **PLOS ONE Journal, Editorial Office**

**Subject:** Submitting a revised version of manuscript and point-by-point response to reviewers

Dear **Academic Editor, PLOS ONE**

Thank you for giving us the opportunity to submit a revised draft of Manuscript ID PONE-D-23-34434R1 entitled "Breastfeeding self-efficacy status and associated factors among postpartum mothers at Hadiya Zone public hospitals, southern Ethiopia"*.* We would like to thank you, the Editor, for handling and facilitating the review process and the reviewers for their precious time in reviewing our paper and providing valuable comments. It was your valuable and insightful comments that led to possible improvements in the current version. The authors have carefully considered the comments and tried our best to address every one of them. We hope the manuscript after careful revisions meet the standards. The authors welcome to respond to any further questions and comments you may have.

Below we provide the point-by-point responses. All modifications in the manuscript have been highlighted within the manuscript.

**Regards,**

**The authors!**

| Responses for academic Editor | | |
| --- | --- | --- |
| No | Academic editor comment/question | **Authors’ response** |
| 1 | The writing is generally clear, but it could be improved by reducing redundancy and providing more specific methodological details | Thank you for your constructive comment. We tried to update methodology and some redundancy is removed from result parts |
| 2 | The study makes a valuable contribution to understanding breastfeeding self-efficacy and its influencing factors in Ethiopia, but it could push the boundaries of knowledge further by exploring new interventions or cultural influences in more depth. | Thank you for your valuable suggestion. We added recommendation in discussion part it for the next researchers to conduct in community level, and consider cultural influences and contextual factors in depth. |
| 3 | The self-reported data in the methodology section introduces potential biases (e.g., recall or social desirability bias), which weakens the reliability of the findings. There is no mention of how data quality was ensured or validated. | Thank you for your valuable concern. We added recall or social desirability bias as limitation.  Data quality control sub-section is added on method part with data quality ensuring issues |
| 4 | There is insufficient detail on the statistical techniques, handling of missing data, or variable selection in the analysis. A more transparent explanation would improve confidence in the findings. | Thank you for your valuable concern. During data cleaning there were no missing data due to careful data collection training and supervision. Concerning variable selection in the analysis, all explanatory variable which have association in bivariate analysis with p value less than or equal 0.25 were entered in to multiple logistic regression model in order to assess the independent predictors of postpartum BFSE status and this type of correction is added in analysis part in method. |
| 5 | While the study highlights important socio-demographic factors, it could provide more concrete suggestions for intervention strategies or programmatic changes in healthcare settings. | Thank you for your comment. Based on our finding we recommended variety of educational interventions to improve mothers' breastfeeding self-efficacy (BFSE). The finding of this cross sectional suggests for educational interventions and equipping healthcare professionals with cultural competency training to enhance their understanding of the beliefs and values of different cultures related to breastfeeding. |
| 6 | The study is significant but could have a larger impact if it offered more innovative or culturally-tailored solutions in the discussion section to improving BFSE. | Thank you for your valuable suggestion and constructive comment. We have tried to add culturally tailored interventions in the discussion part as a recommendation to enhance postpartum BFSE in future research in Ethiopia. |
